# d(−) Lactic Acid-Induced Adhesion of Bovine Neutrophils onto Endothelial Cells Is Dependent on Neutrophils Extracellular Traps Formation and CD11b Expression

**DOI:** 10.3389/fimmu.2017.00975

**Published:** 2017-08-15

**Authors:** Pablo Alarcón, Carolina Manosalva, Ivan Conejeros, María D. Carretta, Tamara Muñoz-Caro, Liliana M. R. Silva, Anja Taubert, Carlos Hermosilla, María A. Hidalgo, Rafael A. Burgos

**Affiliations:** ^1^Laboratory of Inflammation Pharmacology, Faculty of Veterinary Science, Institute of Pharmacology and Morphophysiology, Universidad Austral de Chile, Valdivia, Chile; ^2^Faculty of Sciences, Institute of Pharmacy, Universidad Austral de Chile, Valdivia, Chile; ^3^Faculty of Veterinary Medicine, Institute of Parasitology, Justus Liebig University Giessen, Giessen, Germany

**Keywords:** d(−) lactic acid, neutrophil adhesion, neutrophils, neutrophil extracellular traps, CD11b, ICAM-1

## Abstract

Bovine ruminal acidosis is of economic importance as it contributes to reduced milk and meat production. This phenomenon is mainly attributed to an overload of highly fermentable carbohydrate, resulting in increased d(−) lactic acid levels in serum and plasma. Ruminal acidosis correlates with elevated acute phase proteins in blood, along with neutrophil activation and infiltration into various tissues leading to laminitis and aseptic polysynovitis. Previous studies in bovine neutrophils indicated that d(−) lactic acid decreased expression of L-selectin and increased expression of CD11b to concentrations higher than 6 mM, suggesting a potential role in neutrophil adhesion onto endothelia. The two aims of this study were to evaluate whether d(−) lactic acid influenced neutrophil and endothelial adhesion and to trigger neutrophil extracellular trap (NET) production (NETosis) in exposed neutrophils. Exposure of bovine neutrophils to 5 mM d(−) lactic acid elevated NET release compared to unstimulated neutrophil negative controls. Moreover, this NET contains CD11b and histone H_4_ citrullinated, the latter was dependent on PAD4 activation, a critical enzyme in DNA decondensation and NETosis. Furthermore, NET formation was dependent on d(−) lactic acid plasma membrane transport through monocarboxylate transporter 1 (MCT1). d(−) lactic acid enhanced neutrophil adhesion onto endothelial sheets as demonstrated by *in vitro* neutrophil adhesion assays under continuous physiological flow conditions, indicating that cell adhesion was a NET- and a CD11b/ICAM-1-dependent process. Finally, d(−) lactic acid was demonstrated for the first time to trigger NETosis in a PAD4- and MCT1-dependent manner. Thus, d(−) lactic acid-mediated neutrophil activation may contribute to neutrophil-derived pro-inflammatory processes, such as aseptic laminitis and/or polysynovitis in animals suffering acute ruminal acidosis.

## Introduction

Neutrophils are the first line of defense and the first leukocyte class to arrive at sites of inflammation. Neutrophil recruitment from the blood stream into inflamed tissue is a complex, multistep molecular process mainly mediated by a variety of selectin and integrin proteins that interact with expressed adhesion molecules (e.g., E-selectin, P-selectin, intercellular adhesion molecule 1, and vascular cell adhesion molecule 1) on activated endothelium (1). Once neutrophils arrive to sites of inflammation they display diverse effector mechanisms, including phagocytosis (2), activation of NADPH oxidase [oxidative response (3)], release of granular contents [non-oxidative response (4)], and release of neutrophil extracellular traps (NETs) composed of DNA, histones, microbicidal peptides, and antimicrobial enzymes (5). Many agents induce NET formation, including microorganisms [i.e., bacteria, fungi, viruses (6–9), and parasites (10, 11)], pro-inflammatory factors (6), and metabolic products such as uric acid (12). NET formation leads to a newly appreciated mechanism of cell death, NETosis. The process of NETosis has been linked to Raf/Mek/Erk–MAPK pathway activation (13), PI3K/NADPH oxidase pathway activity (14) and citrullination of histones by PAD4, a mechanism that allows decondensation and unfolding of neutrophil nuclear DNA (15–17). NET formation was described as an effective and ancient antimicrobial mechanism of the host innate immune system resulting in the death of NET-entrapped pathogens and control of invading agents (18). However, excessive NET production, especially surrounding healthy tissues, is harmful to the host and may lead to aseptic inflammatory processes, such as small-vessel vasculitis (19), thrombosis (20), preeclampsia (21), nephritis in systemic lupus erythematosus (22), and metabolic gout disease (12).

Lactic acid is an organic acid found naturally in the enantiomeric forms, l(+) and d(−) lactate. Under physiological conditions in the serum, l(+) lactate predominates in comparison to d(−) lactate owing to anaerobic metabolism of mammalian cells (23). Nonetheless, d(−) lactate concentrations increase under pathophysiological conditions in humans, such as short bowel syndrome [SBS (24, 25)], fatigue syndrome (26), diabetes (27, 28), Crohn’s disease (29), and others. Human patients with d(−) lactic acidosis present with neurological dysfunctions characterized by ataxia, slurred speech, confusion, and weakness (23, 30, 31). In cattle, d(−) lactic acidosis, first reported by Dunlop and Hammond in 1965 (32), is produced by ruminal bacteria during ingestion of large amounts of highly fermentable carbohydrates with low quantities of fiber. This ingestion of excessive carbohydrates is followed by proliferation of lactate-producing microorganisms, such as *Streptococcus bovis* that metabolize carbohydrates to lactate [l(+) and d(−) lactic acid]. This leads to ruminal acidification to pH five or lower. Low ruminal pH has resulted in an imbalance of lactate-consumer and -producer microorganisms, favoring the producer populations (33) and thereby decreasing ruminal pH even further. During acute ruminal acidosis, d(−) lactic acid concentrations in the blood stream have reached levels of 5 mM vs 1.6 mM of l(+) lactic acid (34). This can be explained because, d(−) lactic acid is rapidly absorbed but more slowly metabolized than l(+) lactic acid by bovine tissues (35). Thus, only d(−) lactic acid blood concentrations is increased in cattle with ruminal acidosis, while l(+) lactic acid remains in the physiological range (23, 32, 34), suggesting that d(−) lactic, but not l(+) form, is responsible for the clinical findings observed during ruminal acidosis. Animals with acute ruminal acidosis induced by oral oligofructose overloads had elevated acute phase proteins (36). This suggested that pro-inflammatory responses in these animals altered their locomotor apparatuses, which presented as acute aseptic laminitis (37) and polysynovitis (38). Both pathological entities are classified as aseptic inflammatory diseases, characterized by massive leukocyte infiltration of the lamellae (37, 39) and neutrophil recruitment to synovial fluid of affected animals (38). Interestingly, neutrophils exposed to lactic acid had decreased intracellular pH and reduced surface L-selectin (40). In addition, decreased L-selectin expression in bovine neutrophils was observed in experimentally induced bovine ruminal acidosis, which has been implicated in the L-selectin shedding mechanism (41). Besides, d(−) lactic induced L-selectin shedding and increased expression of CD11b in bovine neutrophils after stimulation with 6 mM of d(−) lactic acid (42). Taken together, these data suggested that neutrophils, under ruminal acidosis d(−) lactic acid conditions, may have induced neutrophil adhesion to vascular endothelium. In this study, we demonstrated not only that d(−) lactic acid induced neutrophil adhesion onto vascular endothelial sheets under physiological flow conditions but also that neutrophil–endothelium adhesion was a NET-dependent mechanism.

## Materials and Methods

### Animals

Four dairy heifers with body weights of 280–310 kg from the University Austral herd were used in all experiments. Heifers were maintained on an *ad libitum* grass diet with grain supplementation. All experiments were conducted in strict accordance with protocols approved by the ethical committee of the Universidad Austral de Chile (permit number: 217/2015) and according to the current Chilean Animal Protect Laws.

### Primary Bovine Umbilical Vein Endothelial Cell (BUVEC) Isolations

Primary BUVEC was isolated from bovine umbilical cords, as previously shown (43). Umbilical cords obtained from calves born by cesarean section were kept at 4°C in 0.9% Hank’s Balanced Salt Solution–HEPES (HBSS–HEPES) buffer (pH 7.4, Gibco, Thermo Fisher Scientific, Waltham, MA, USA), supplemented with 500 U/ml penicillin (Sigma-Aldrich, St. Louis, MO, USA) and 50 mg/ml streptomycin (Sigma-Aldrich, St. Louis, MO, USA) for 1 h. After clamping one end of the umbilical cord veins, 0.025% type II collagenase (Worthington Biochemicals Corporation, Lakewood, NJ, USA) in HBSS (Gibco, Thermo Fisher Scientific, Waltham, MA, USA) was infused into the vein lumens. The opened ends of the veins were closed and then incubated at 37°C and 5% CO_2_ for 20 min. Thereafter, the veins were gently massaged and the collagenase solutions were collected in 50 ml plastic tubes (Nunc, Thermo Fisher Scientific, Waltham, MA, USA) containing 1 ml FCS (Gibco, Thermo Fisher Scientific, Waltham, MA, USA) to inactivate the collagenase. The venous lumens were washed two times with RPMI 1640 medium (Gibco, Thermo Fisher Scientific, Waltham, MA, USA). The washes were pooled and centrifuged at 400 × *g* for 10 min. The cell pellets were resuspended in complete ECM (Sciencell Research Laboratories, Carlsbad, CA, USA), cells were plated in 25 cm^2^ plastic tissue culture flasks (Nunc Thermo Fisher Scientific, Waltham, MA, USA) and incubated at 37°C and 5% CO_2_ until reaching confluence.

### Isolation of Neutrophils

Blood was collected by jugular vein puncture, and neutrophils were isolated as described previously (44). Briefly, following collection into acid citrate dextrose-lined collection tubes (Becton Dickinson, USA), blood specimens were gently rocked for 5 min (Nutator, Becton Dickinson, Franklin Lakes, NJ, USA) and then centrifuged at 1,000 × *g* at 20°C for 20 min. The plasma and buffy coat layers were aspirated, and remaining red blood cells and neutrophil pellets were suspended in HBSS. Red blood cells were removed by flash hypotonic lysis using a cold phosphate-buffered water solution (5.5 mM NaH_2_PO_4_, 8.4 mM HK_2_PO_4_, pH 7.2). Once returned to isotonicity using a hypertonic phosphate buffer solution (5.5 mM NaH_2_PO_4_, 8.4 mM HK_2_PO_4_, 0.46 M NaCl, pH 7.2), the samples were centrifuged at 600 × *g* at 20°C for 10 min. The neutrophil pellets were washed thrice with HBSS. Neutrophil viabilities were determined by trypan blue assays and were never less than 97% by light microscopy. Purity was at least 94%, as assessed by flow cytometry analyses using forward-scatter vs side-scatter dot plot.

### Neutrophil Adhesion Assays under Physiological Flow Conditions

Bovine umbilical vein endothelial cell was cultivated on coated Thermanox^®^ coverslips (22 mm × 60 mm; Nunc, Thermo Fisher Scientific, Waltham, MA, USA) with 10 µg/ml bovine fibronectin (#F1141 Sigma-Aldrich, St. Louis, MO, USA) until reaching confluence. BUVEC monolayers on Thermanox^®^ coverslips were mounted in a parallel plate flow chamber allowing constant and uniform laminar flow fields, as previously described (45, 46). Pre-warmed (37°C) pH buffer (140 mM NaCl, 10 mM glucose, 1 mM KCl, 1 mM CaCl_2_, 1 mM MgCl_2_, and 20 mM HEPES) was perfused over the BUVEC for 2–3 min to remove soluble factors and cell debris. Subsequently, neutrophil suspensions were perfused into the systems at flow rates that resulted in a physiological constant wall shear stress of 1.0 dyn/cm^2^ (syringe pump sp 100i; World Precision Instruments, Berlin, Germany), corresponding to the flow rates of small venous vessels. Interactions between BUVEC and neutrophils were visualized using a phase-contrast microscope (Olympus CKX41, Miami, FL, USA) and video (Optikam PRO 5 Digital camera, Ponteranica, BG, Italy) using AMCap v 9.21 software and an Xvid MPEG-4 codec video compressor. After 10 min, quantification of neutrophil adhesion was performed microscopically by determining the number of adherent cells in 10–20 randomly selected vision fields with modifications as published by Maksimov et al. (47). In each adhesion assay, neutrophils of least four different animals were used. For all experiments, primary BUVEC isolates had been passaged at least three, but no more than seven times, as described in Hermosilla et al. (48). In order to discard a possible effect of pH [5.7 in the media with 5 mM d(−) lactic acid], we used HCl.

### Quantification of NETs

Neutrophil extracellular traps were quantitated by measuring the amount of extracellular DNA that was stained by the cell-permeant fluorescent dye PicoGreen^®^ (Invitrogen, Carlsbad, CA, USA). 1 × 10^6^ neutrophils were incubated with different concentrations of d(−) lactic acid (0.1, 0.7, 2.0, and 5.0 mM), 1 µM of ionomycin or vehicle for 30 min. Micrococcal nucleases were added (5 U/well, New England Biolabs, Ipswich, MA, USA) (15 min, 37°C). Samples were centrifuged (300 × *g*, 5 min). The supernatants were transferred (100 ml per 96-well) and PicoGreen^®^ (50 ml/well, diluted 1:200 in 10 mM Tris/1 mM EDTA) was added. NET formation was determined by spectrofluorometric analysis (484 nm excitation/520 nm emission) using an automated reader (Varioskan Flash; ThermoFisher Scientific, Waltham, MA, USA) as described elsewhere (10).

### Visualization of NET and Detection of CD11b and Citrunillated 3 Histone H_4_ in NET-Like Structures

A total of 1 × 10^6^ neutrophils were incubated with 1 µM Ar-c155858, 200 µM of Cl-amidine or vehicle (0.01% dimethyl sulfoxide) for 60 min at 37°C. Next, cells were stimulated with 5 mM of d(−) lactic acid with 90 U DNase I (PanReac AppliChem, Darmstadt, Germany) or with vehicle (0.01% ethanol) for 30 min at 37°C. Next, neutrophils were fixed with 2% paraformaldehyde solution for 20 min at room temperature (RT). The cells were treated with blocking buffer (4% of non-fat milk, 1% BSA, and 0.05% Tween-20 in PBS) for 2 h at RT and incubated with either anti-CD11b antibody (#MCA1425, ABD Serotec, Raleigh, NC, USA), or anti-H_4_ citrullinated 3 antibody (#07-596, Merck Millipore, Darmstadt, Germany) overnight at 4°C. Neutrophils were washed with PBS three times and incubated with goat anti-rabbit IgG-Alexa 405 (#A31556, Thermo Scientific, Waltham, MA, USA), or goat anti-mouse IgG-Alexa 635 (#A31575, Thermo Fisher Scientific, Waltham, MA, USA) for 2 h at RT. Nuclei were visualized with PicoGreen^®^ 1:200 dilution (#P7589, Thermo Fisher Scientific, Waltham, MA, USA). The quantification of NETs was performed using the formula (Nets for field/total Neutrophils) × 100 (49) and depicted as fold of control.

### Scanning Electron Microscopy (SEM) Analyses

We treated 250 × 10^3^ neutrophils with 5 mM of d(−) lactic acid or vehicle for 30 min. Cells were fixed in 2.5% glutaraldehyde in 0.1 M cacodylate buffer for 15 min and washed gently with 0.1 M cacodylate buffer (all Merck, Darmstadt, Germany). The cells were then post-fixed in 1% osmium tetroxide (Merck, Darmstadt, Germany) diluted in 0.1 M cacodylate buffer, washed three times in distilled water, dehydrated in ascending ethanol concentrations, critical point dried by CO_2_ treatment and sputtered with gold. Specimens were examined using a Philips XL30 scanning electron microscope at the Institute of Anatomy and Cell Biology, Justus Liebig University Giessen, Germany.

### Quantification Neutrophil MCT mRNA Expression with Real-time PCR

Total RNA specimens were isolated from 10 × 10^6^ neutrophils per subject using EZNA^®^ Total RNA Kits (E.Z.N.A.; Promega, Madison, WI, USA). Samples were treated with Turbo DNase-Free^®^ (Thermofisher Scientific, Waltham, MA, USA). For the cDNA synthesis reaction, 700 ng of total RNA was reverse transcribed using Affinity Script^®^ QPCR cDNA Synthesis Kits (Agilent Technologies, Cedar Creek, TX, USA). Real-time PCR assays were performed using SYBR Green^®^ qPCR Master mix reagents (Fermentas Life Sciences, Waltham, MA, USA) and primers specific for bovine monocarboxylate transporter 1 (MCT1), MCT2, MCT3, MCT4, and housekeeping ribonucleoprotein s9 (RPS9). The primers used for the PCR were as follows: MCT1 forward 5′CGCCGCGAGCCGCGTATAA 3′ reverse 5′CCTCCAACTGCTGGTGGCATTGT3′; MCT2 forward 5′CCACCCAGTGCCGGAGA CCA 3′, reverse 5′TCCCGTGTCTAAGGTTGCCCAGG 3′; MCT3 forward 5′GAGGCT GTGGCTGTGCTCATCG 3′ reverse 5′GATCTCGTAGTTCTTGAGCGCGT CC 3′; MCT4 forward 5′ATCCAGCAAGCCCTCCCTTCCC 3′, reverse 5′CCATGGCCAGGAG GGCTGATTC T3′, RSP9 forward 5′GCTGACGCTG GATGAGAAAGACCC 3′, and reverse 5′ATCCAGCACCCCGATAC GGACG 3′. We used the following conditions on a Mx3000P qPCR System (Agilent Technologies, Santa Clara, CA, USA): (1) 1 cycle of 95°C for 10 min, (2) 35 cycles of 95°C for 30 s, 60°C for 60 s, and 72°C for 60 s, and (3; dissociation curve) 95°C for 1 min, 60°C for 30 s, and 95°C for 30 s.

### Immunofluorescence of MCT1, MCT4, and CD147

For immunofluorescence visualization of MCTs, 2.5 × 10^5^ neutrophils were incubated in a 3% paraformaldehyde/0.19 M sucrose solution for 15 min and then transferred to slides using cytospin. Slides were washed with PBS and then neutrophils were permeabilized with 100 µM digitonin (Sigma-Aldrich, St. Louis, MO, USA) for 10 min. Slides were washed with PBS, treated with blocking buffer for 1 h, and incubated with anti-MCT1- (#bs-1569R, BIOSSUSA, Woburn, MA, USA), anti-MCT4- (#bs-2698R, BIOSSUSA, Woburn, MA, USA), and anti-CD147- (EMMPRIN H-200: sc-13976, Santa Cruz Biotechnology, Dallas, TX, USA) specific antibodies, overnight. Next, the slides were incubated with goat anti-rabbit IgG-Alexa Fluor 488 (#A11034, Thermofisher Scientific, Waltham, MA, USA) for 2 h. To visualize nuclei, slides were Hoechst 33342 (#H3570, Invitrogen, Thermo Fisher Scientific, Waltham, MA, USA) stained at 2.5 µg/ml for 20 min. Images were acquired with an OLYMPUS^®^ Fluoview 1000 confocal microscope.

### Immunoblot

10 × 10^6^ neutrophils were lysed in lysis buffer (50 mM Tris–HCl, pH 8.0; 150 mM NaCl, 1% of NP-40, and 10 µg/ml of protease inhibitors: leupeptin, aprotinin, and pepstatin). Total protein (80 µg) were separated on 12% SDS-PAGE gels and transferred onto nitrocellulose membranes. Membranes were then blocked in TBS buffer (0.1% Tween-20 and 5% non-fat dry milk) for 2 h at RT, washed with TBS-Tween 0.1% (10 mM Tris–HCl, 68 mM NaCl, and 0.1% Tween-20), and incubated with MCT1 (#bs-1569R, BIOSSUSA, Woburn, MA, USA), MCT2 (#ab182178, Abcam, Cambridge, UK), MCT3 (#ab60333, Abcam, Cambridge, UK), MCT4 (#bs-2698R, BIOSSUSA, Woburn, MA, USA), and CD147 (EMMPRIN H-200: sc-13976, Santa Cruz Biotechnology, Dallas, TX, USA) antibodies overnight at a 1:2,000 dilution at 4°C. Membranes were incubated with HRP-conjugated secondary antibody (#sc-2770, Santa Cruz Biotechnology, Dallas, TX, USA) for 2 h at RT, and visualized using an enhanced chemoluminescence system (Thermofisher Scientific, Waltham, MA, USA). Molecular weights of proteins were determined based on the mobility of pre-stained standards of known molecular weight. For control experiments, MCT1, MCT4, and CD147 antibodies were removed by incubation with a stripping solution (100 mM 2-mercaptoethanol; 2% SDS; 62.5 mM Tris–HCl, pH 6.7) for 1 h at 50°C with agitation, followed by several washes with TBS-Tween 0.1%. Membranes were then incubated with anti-β-actin antibodies (#A1978, Sigma-Aldrich, St. Louis, MO, USA).

### Determination of l(+) and d(−) Lactate Concentrations in Bovine Neutrophils by HPLC

We treated 5 × 10^6^ neutrophils with 1 µM of Ar-c1555858 (MCT1 inhibitor) or vehicle (0.01% DMSO) for 60 min at 37°C and later stimulated either with 2 mM of l(+) lactic acid or 2 mM of d(−) lactic acid for 30 min at 37°C. Cells were centrifuged at 800 × *g* for 6 min. Cell pellets were lysed with liquid nitrogen and resuspended in 500 µl of 1 mM CuSO_4_ (mobile phase). Lysed cells were centrifuged at 800 × *g* for 6 min and supernatants (500 µl) were loaded into Amicon^®^ Ultra-4 3K (Merck Millipore, Darmstadt, Germany) tubes and centrifuged at 75,000 × *g* for 40 min at 4°C (50). The filtrates were concentrated in a SpeedVac Concentrator (Savant^®^ SPD131DDA, Thermo Fisher) for 90 min at 45°C and 1.5 atmospheres of pressure. Finally, the concentrates were resuspended in 200 µl of 1 mM CuSO_4_, as reported elsewhere (51). For calibration curves, 2–400 µM aliquots of l(+) and d(−) lactic acid were used. Twenty-microliter samples were analyzed by HPLC using a cationic exchange column, Astec CLC-D (15 cm × 4.6 mm, Sigma-Aldrich, St. Louis, MO, USA), at a 1.2 ml/min flow rate at 30°C. The detection wavelength was set at 254 nm (52) using diode array HPLC, Elite la Chrom (VWR-Hitachi, PA, USA).

### Endothelium Cell Death Assays

Primary BUVEC was seeded onto 96-well plates at a final concentration of 20,000 cells/ml. Briefly, BUVECs were treated with 5 mM of d(−) lactic acid, vehicle (pH buffer), or with supernatants from neutrophils exposed to 5 mM of d(−) lactic acid, DNase I, or 0.01% vehicle (ethanol) for 30 min at 37°C. As a positive control for cell death, cells were treated with 0.2% Triton X-100 diluted in pH buffer at 37°C for 30 min. Cells were incubated with 5 µM propidium iodide (PI; Invitrogen, Carlsbad, CA, USA) diluted in pH buffer for 15 min. The PI signals were detected with a fluorescence multiplate reader (Varioskan^®^, Thermo Scientific) at 530 nm excitation/620 nm emission wavelengths.

### Statistical Analyses

All results presented here in bar graphs or dot plots illustrate mean ± S.E.M. of five independent experiments obtained from five heifers. One-way analysis of variance calculations were performed, and Dunnett’s multiple comparison tests were applied, with significance considered for *P*-values less than 0.05. Statistical analyses were performed using GraphPad Prism^®^ (v 5.0; Graphpad Software, USA).

## Results

### d(−) Lactic Acid-Induced Bovine Neutrophil Adhesion onto Endothelial Cell Sheets

**Figure 1:**
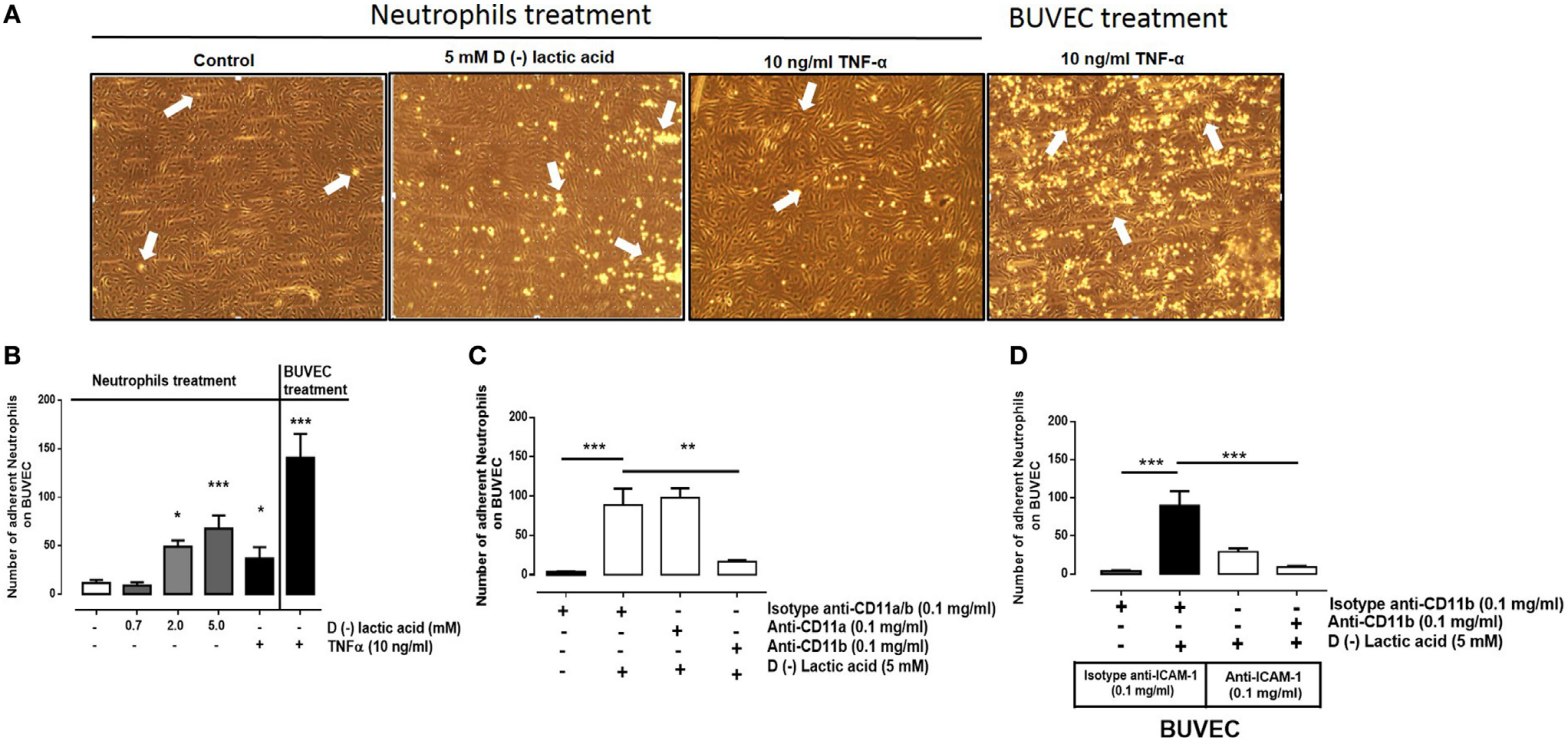
d(−) lactic acid-induced adhesion of neutrophils on vascular endothelium was CD11b/ICAM-1-dependent. **(A)** Representative images of adhesions under flow conditions with neutrophils treated with TNFα (10 ng/ml), d(−) lactic acid (5 mM), or vehicle on bovine umbilical vein endothelial cell (BUVEC) monolayers. As positive control of experiment, we perfused neutrophils without treatment onto BUVEC stimulated with TNFα (10 ng/ml) for 24 h. **(B)** Mean ± S.E.M. of adherent neutrophils on BUVEC, treated with different concentrations of d(−) lactic acid (0.7, 2.0, and 5.0 mM), TNFα (10 ng/ml), or vehicle for 30 min, *n* = 5. **(C)** Mean ± S.E.M. of neutrophil adhesions after pre-incubation with 5 mM d(−) lactic acid concomitant with anti-CD11a, anti-CD11b, or isotype control antibodies for 30 min and perfused onto a monolayer of BUVECs under flow conditions, *n* = 5. **(D)** Mean ± S.E.M. of adherent neutrophils on BUVEC sheets, co-treated with 5 mM d(−) lactic acid and anti-CD11b or isotype controls. In parallel, BUVEC was incubated with anti-ICAM-1 or isotype controls. Both treatments were for 30 min, and then neutrophils were perfused onto BUVEC. *n* = 5, *P < 0.05; **P < 0.01; ***P < 0.001 using Dunnett’s multiple comparison test.

We previously demonstrated that d(−) lactic acid increased CD11b expression and induced L-selectin shedding in bovine neutrophils (42), suggesting that d(−) lactic acid induced neutrophil adhesion to endothelial cells. Here, this was evaluated with neutrophil adhesion assays onto BUVEC monolayers under physiological flow conditions in parallel plate flow chamber systems. Neutrophils were stimulated with increasing concentrations of d(−) lactic acid (0.7–5.0 mM) for 30 min and then cells were perfused onto BUVEC monolayers. d(−) lactic acid at 5 mM concentrations significantly increased neutrophil adhesions onto vascular endothelial sheets (Figures 1A,B). TNFα (10 ng/ml) stimulation of neutrophils (30 min) and BUVEC monolayers (24 h) served as positive controls, as previously described (48). We discard an effect of pH (5.7) using HCl, which was not able to induce bovine neutrophil adhesion (Figure S1 in Supplementary Material). Next, neutrophils were treated with d(−) lactic acid concomitant with anti-CD11a- and anti-CD11b-specific antibodies (Figure 1C). When co-stimulated with anti-CD11b antibodies, significant reductions of neutrophil adhesions onto BUVEC monolayers were observed. Conversely, co-stimulation with anti-CD11a antibodies did not alter lactic acid-induced neutrophil adhesions. CD11b has been demonstrated to promote amphotericin-induced adhesion, independent of CD11a (53). Furthermore, when neutrophils were co-stimulated with 5 mM d(−) lactic acid and anti-CD11b antibodies and then perfused onto anti-ICAM-1-blocked endothelial sheets, neutrophil adhesion decreased more than when neutrophils were only treated with 5 mM d(−) lactic acid and isotype antibodies (Figure 1D). Together, these results suggested that d(−) lactic induced significant neutrophil adhesion reaction onto vascular endothelium, which was a CD11b/ICAM-1-dependent mechanism.

### d(−) Lactic Acid-Induced Bovine NET Extrusion

**Figure 2:**
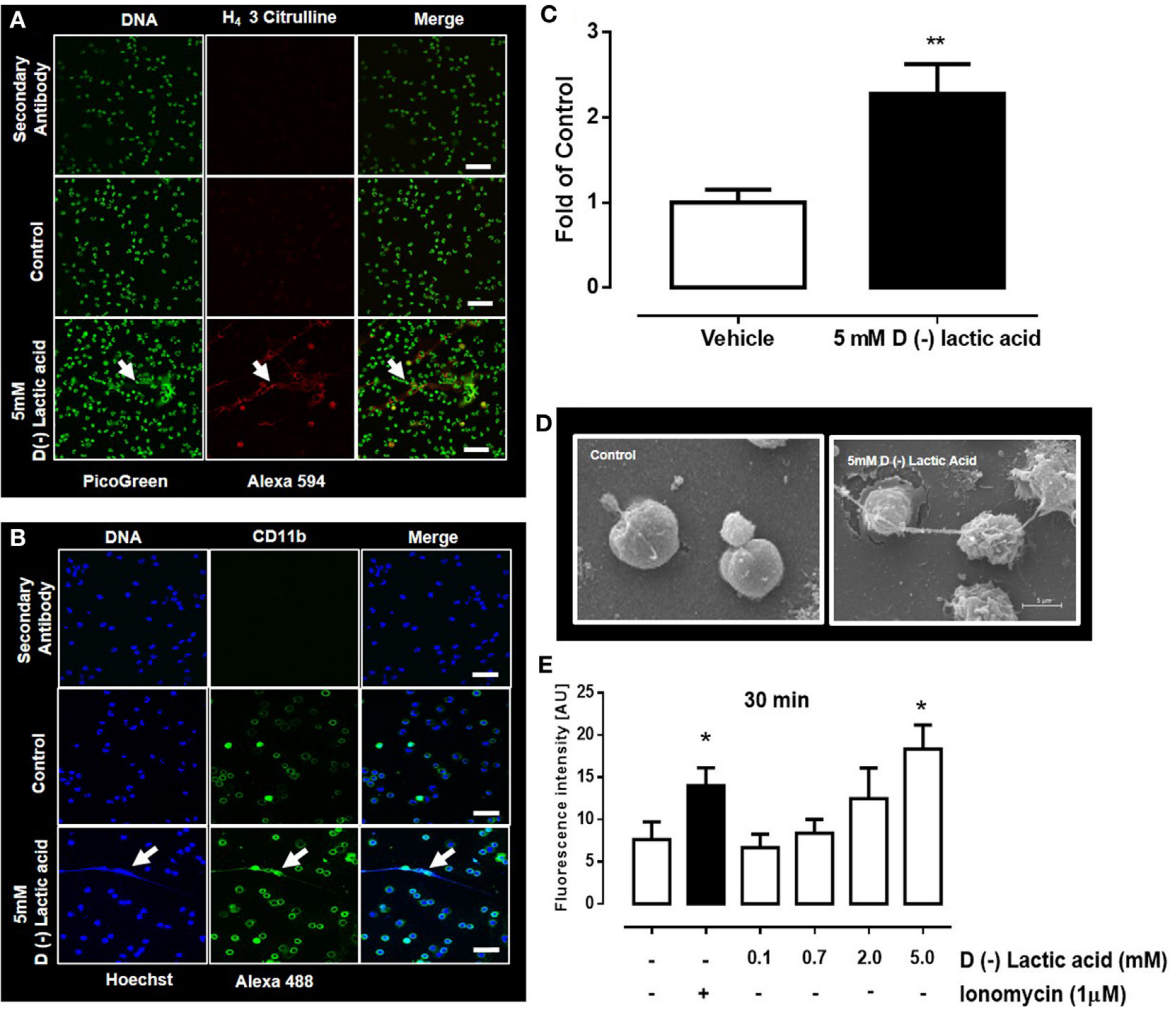
d(−) lactic acid-induced neutrophil extracellular trap (NET) formation. **(A,B)** Immunofluorescent images of bovine neutrophils treated with 5 mM d(−) lactic acid for 30 min using anti-H_4_ citrullinated 3 **(A)** and CD11b **(B)** markers, with picogreen and Hoechst 33342 as DNA staining probes, respectively; representative images of five independent experiments, scale bars: 60 µm for **(A)** and 50 µm for **(B)**. **(C)** Fold of control [(number of NETs/number of neutrophils) × 100]. **(D)** Images from scanning electronic microscopy of neutrophils treated with d(−) lactic acid, scale bar: 5 µm, representative images from three independent experiments. **(E)** Extracellular DNA quantifications of supernatants from neutrophils treated with different concentrations of d(−) lactic acid (0.1–5.0 mM), ionomycin (positive control) or vehicle using picogreen to stain DNA, *n* = 5. **P < 0.01 using Student’s *t-*test and *P < 0.05 using Dunnett’s multiple comparison test. Arrow indicates NET structure.

The mechanism of NET formation was originally described by Brinkmann et al. (5). In this mechanism, neutrophils release granules composed of nuclear DNA fibers coated with histones (H_1_, H_2A_/H_2B_, H_3_, H_4_) and proteolytic enzymes. These fibers ensnare microbes and, thus, control microbial infection of the host (54). Bovine neutrophils stimulated with vital *Eimeria bovis* sporozoites efficiently trigger NET formation and increased CD11b surface expression (10). Consistent with these findings, bovine neutrophils exposed to 5 mM d(−) lactic acid for 30 min resulted in extrusion of NET-like structures (Figure 2C) that were positive for H_4_ citrullinated (Figure 2A) and CD11b (Figure 2B). The presence of extracellular NET structures were confirmed by SEM analyses in which thin fibers released from lactic acid-treated neutrophils were detected (Figure 2D). Quantification of NET by DNA release showed that stimulation with 5 mM of d(−) lactic acid for 30 min significantly induced the release of extracellular DNA (Figure 2E).

### MCT Expression and Cellular Lactate Transport

**Figure 3:**
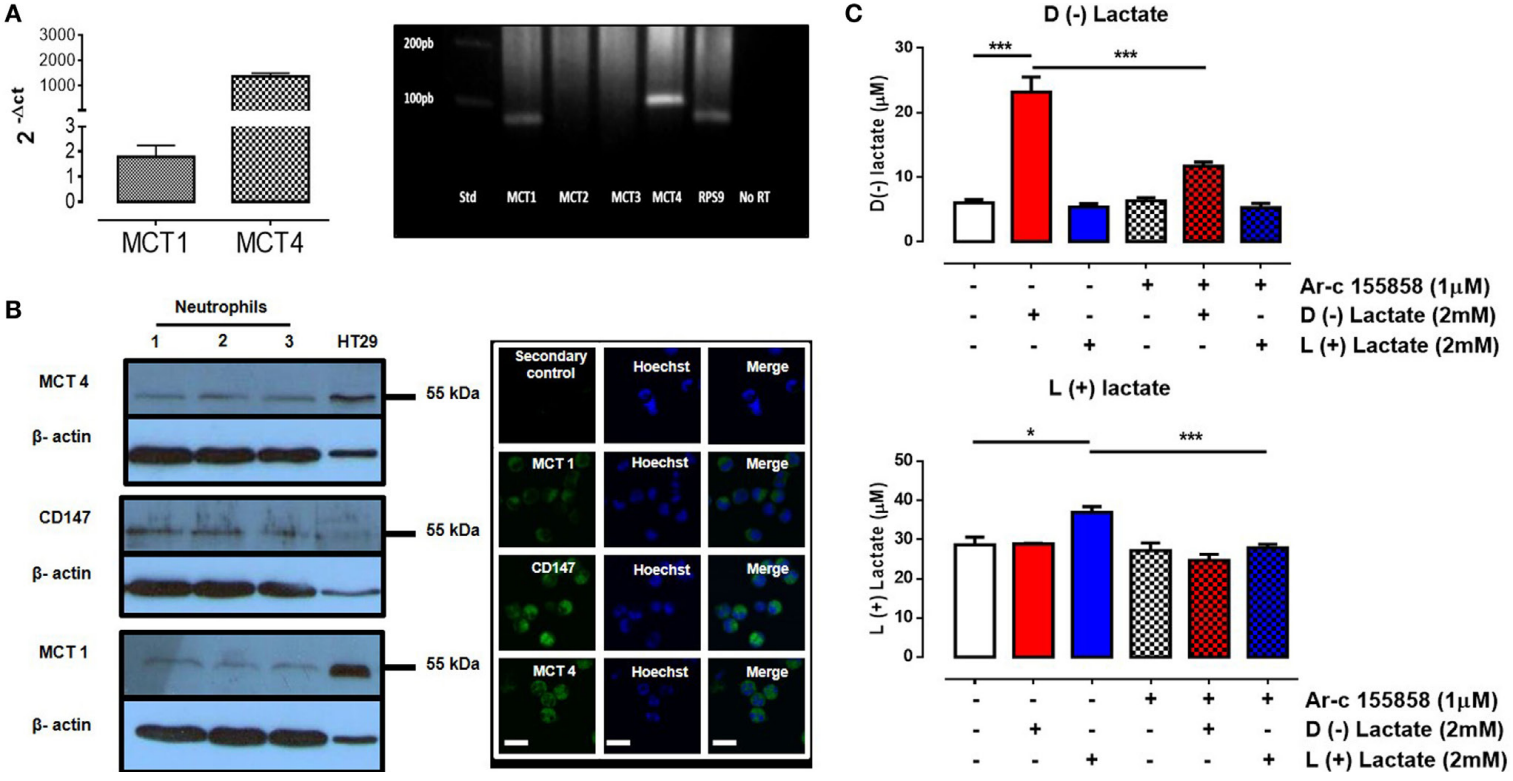
Uptake of d(−) lactate was mediated by monocarboxylate transporter 1 (MCT1). **(A)** Relative mRNA levels of MCT1, MCT2, MCT3, and MCT4 were quantified by qRT-PCR from bovine neutrophils normalized with ribonucleoprotein S9 (housekeeping) and levels were visualized on a 1% agarose gel. **(B)** Western blots of bovine neutrophil lysates were performed using HT29 levels as positive controls. Immunofluorescence of MCT1, 4, and CD147 in bovine neutrophils was visualized using Hoechst to stain DNA, scale bar: 40 μm. **(C)** Intracellular levels of d(−) and l(+) lactate was measured by HPLC in bovine neutrophils that were pre-treated with MCT1 inhibitor (Ar-c155858) or vehicle and then stimulated with d(−) and l(+) lactic acid for 30 min, *n* = 5, *P < 0.05, ***P < 0.001 using Dunnett’s multiple comparison test.

Monocarboxylates, such us pyruvate, lactate, and ketone bodies (d-beta-hydroxybutyrate and acetoacetate), are transported across the plasma membrane through MCTs [e.g., SLC16 (55, 56)]. The monocarboxylate family has 14 members, but only four of them (MCT1–4) bi-directionally transport monocarboxylates coupled to protons (57). In human immune cells, such as leukocytes, MCT1, MCT2, and MCT4, proteins have been detected in granulocytes, lymphocytes, and monocytes (58). These transporters were expressed concomitantly with ancillary cell surface glycoproteins such as CD147 (also known as OX-47, EMMPRIN, HT7, and/or basignin). CD147 is a chaperone protein that facilitates cell surface expression of MCT1 and 4 (59). In bovine neutrophils, RPS9, MCT1, and MCT4 mRNAs were expressed, while MCT2 and MCT3 were absent (Figure S2 in Supplementary Material). MCT4 expression levels were 1,000-fold lower than MCT1 levels (Figure 3A). Expressions of MCT1 and MCT4 gene products in bovine neutrophils were corroborated by western blot and immunofluorescence analyses (Figure 3B). Presence of the chaperone protein CD147 on neutrophils was also observed (Figure 3B). Monocarboxylate transport inside neutrophils was measured by assaying intracellular contents with HPLC analyses. Neutrophils were stimulated with either 2 mM of l(+) or d(−) lactic acid or vehicle (HBSS) for 30 min. Stimulation with either enantiomer increased intracellular monocarboxylate concentrations when compared with vehicle controls. However, uptake of l(+) or d(−) lactic acid was reduced by pre-treatment of neutrophils with Ar-c155858 [a selective and specific pharmacological inhibitor of MCT1 (60); Figure 3C (top and bottom panels)]. Together, these results demonstrated that MCT1 and MCT4, and CD147 gene products were expressed in bovine neutrophils. The data suggested that MCT1 facilitated l(+) or d(−) lactate intracellular transport into these cells.

### d(−) Lactic Acid-Induced Neutrophil Adhesion Was NET- and PAD4-Dependent

**Figure 4:**
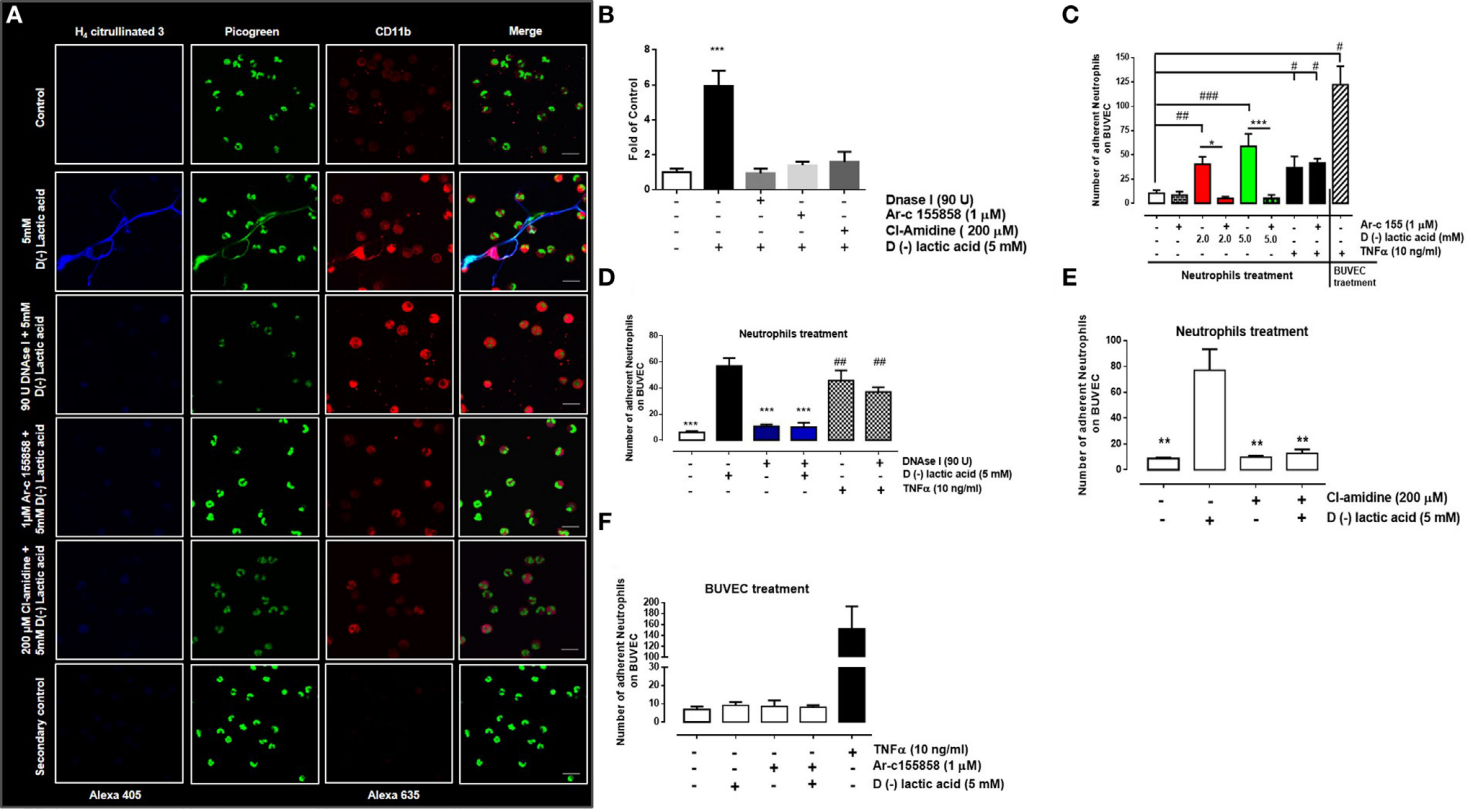
PAD4 activation regulates d(−) lactic acid-induced neutrophil extracellular trap (NET) formation and subsequent adhesion. **(A)** Immunofluorescence of bovine neutrophils pre-treated with monocarboxylate transporter 1 inhibitor (Ar-c155858), PAD4 inhibitor (Cl-amidine), or vehicle (0.01% DMSO) for 60 min, followed by stimulation with either 5 mM d(−) lactic acid or vehicle concomitant with DNase I (90 U) or controls for 30 min. Neutrophils were probed with anti-H_4_ citrullinated 3/alexa 405, CD11b/alexa 635, and PicoGreen as a probe for DNA; representative images from three independent experiments, scale bar: 50 µm. **(B)** Fold of control [(number of NETs/number of neutrophils) × 100] from neutrophils treated with 1 µM Ar-c155858, 200 µM Cl-amidine or vehicle for 1 h and stimulated with 5 mM of d(−) lactic acid or vehicle (control) together or not with DNAse I (90 U) for 30 min, *n* = 3. ***P < 0.001 compared with control, using Dunnett’s multiple comparison test. **(C)** Mean ± S.E.M. of adherent neutrophils pre-treated with Ar-c155858 and stimulated with a gradient of d(−) lactic acid (0.7; 2.0, and 5.0 mM), TNFα (10 ng/ml), or vehicle for 30 min. bovine umbilical vein endothelial cells (BUVECs) treated with TNFα (10 ng/ml) for 24 h served as positive controls for adhesion, *n* = 5. **(D)** Mean ± S.E.M. of adherent neutrophils treated with 5 mM d(−) lactic acid or TNFα (10 ng/ml) and co-treated with DNAse I (90 U) or control solution for 30 min under flow conditions. TNF-α (10 ng/ml) treated cells were used as positive control, *n* = 5, ^#^*P* < 0.05; ^##^*P* < 0.01; ^###^*P* < 0.001 compared with controls, *P < 0.05 and ***P < 0.001 compared with d(−) lactic acid-treated samples, using Dunnett’s multiple comparison test. **(E)** Mean ± S.E.M. of adherent neutrophils pre-treated with PAD4 inhibitor (Cl-amidine) or vehicle for 60 min then stimulated with either 5 mM d(−) lactic acid or vehicle for 30 min under flow conditions, *n* = 5, ***P < 0.001, using Dunnett’s multiple comparison test. **(F)** Mean ± S.E.M. of adherent neutrophils onto BUVEC pre-treated with Ar-c155858 and then stimulated with 5 mM d(−) lactic acid, *n* = 5.

The role of MCT1 activity in NET formation by neutrophils was assessed. Neutrophils were incubated with 1 µM of Ar-c155858 to inhibit endogenous MCT1 and then stimulated with 5 mM d(−) lactic acid. Co-treatment with these agents resulted in significant reduction of NET formation in comparison to d(−) lactic acid treatment alone (Figure 4A). Similarly, treatment with a PAD4 inhibitor (200 µM, Cl-amidine) and DNase I (90 U) reduced neutrophil NET formation, suggesting that d(−) lactic acid-induced NET formation was PAD4- and MCT1-dependant (Figures 4A,B). Furthermore, the role of MCT1 on neutrophil adhesion under continuous flow conditions onto endothelium was assessed. Bovine neutrophils treated with Ar-c155858 to inhibit MCT1 significantly decreased neutrophil adhesion of cells that were previously exposed to 2 and 5 mM d(−) lactic acid (Figure 4C). TNFα is a pro-inflammatory cytokine that has been shown to stimulate adhesion of neutrophils to endothelium through activation of the β-integrin, CD11b/CD18 (61), and NET formation (15). TNFα-induced effects on neutrophils are thought to be mediated through activation of TNF receptors (62) and not through MCT transporter mechanisms. To determine whether Ar-c155858 specifically inhibited d(−) lactic acid-induced neutrophil adhesion, the role of MCT in TNFα-induced neutrophil adhesion was evaluated. Interestingly, Ar-c155858 treatment did not inhibit TNFα-mediated neutrophil adhesion on bovine endothelial sheets in parallel flow chamber assays (Figure 4C). Stimulation of BUVEC monolayers with TNFα for 24 h was used as a positive control. Co-stimulation with DNase I and d(−) lactic acid treatments also significantly decreased neutrophil adhesions onto bovine endothelial sheets. Conversely, DNase I treatments did not alter TNFα-induced neutrophil adhesions onto BUVEC monolayers (Figure 4D).

The role of PAD4 in d(−) lactic acid-induced neutrophil adhesion was evaluated. Adhesion was significantly inhibited after Cl-amidine pre-treatment, prior to stimulation with d(−) lactic acid (Figure 4E). To evaluate whether d(−) lactic acid BUVEC stimulation increased neutrophil adhesions, untreated neutrophils were perfused onto endothelial sheets that had been previously treated with Ar-c155858 or corresponding vehicle (0.01% DMSO), and then cells were stimulated with 5 mM of d(−) lactic acid. Neutrophil adhesions onto endothelial sheets were not observed when compared to positive controls (i.e., TNFα; Figure 4F). Together these results suggested that neutrophil adhesions onto bovine endothelial sheets were dependent on d(−) lactic acid uptake through MCT1 and that NET formations were mediated by PAD4. Neutrophils stimulated with d(−) lactic acid increased adhesions to endothelia that were not recapitulated by direct endothelial stimulations. Moreover, DNase I and Ar-c155858 treatments specifically inhibited d(−) lactic acid-induced neutrophil adhesions.

### d(−) Lactic Acid-Induced Cell-Free DNA (cf-DNA) Release from Neutrophils That Influenced Adhesion with Endothelial Cell Sheets

**Figure 5:**
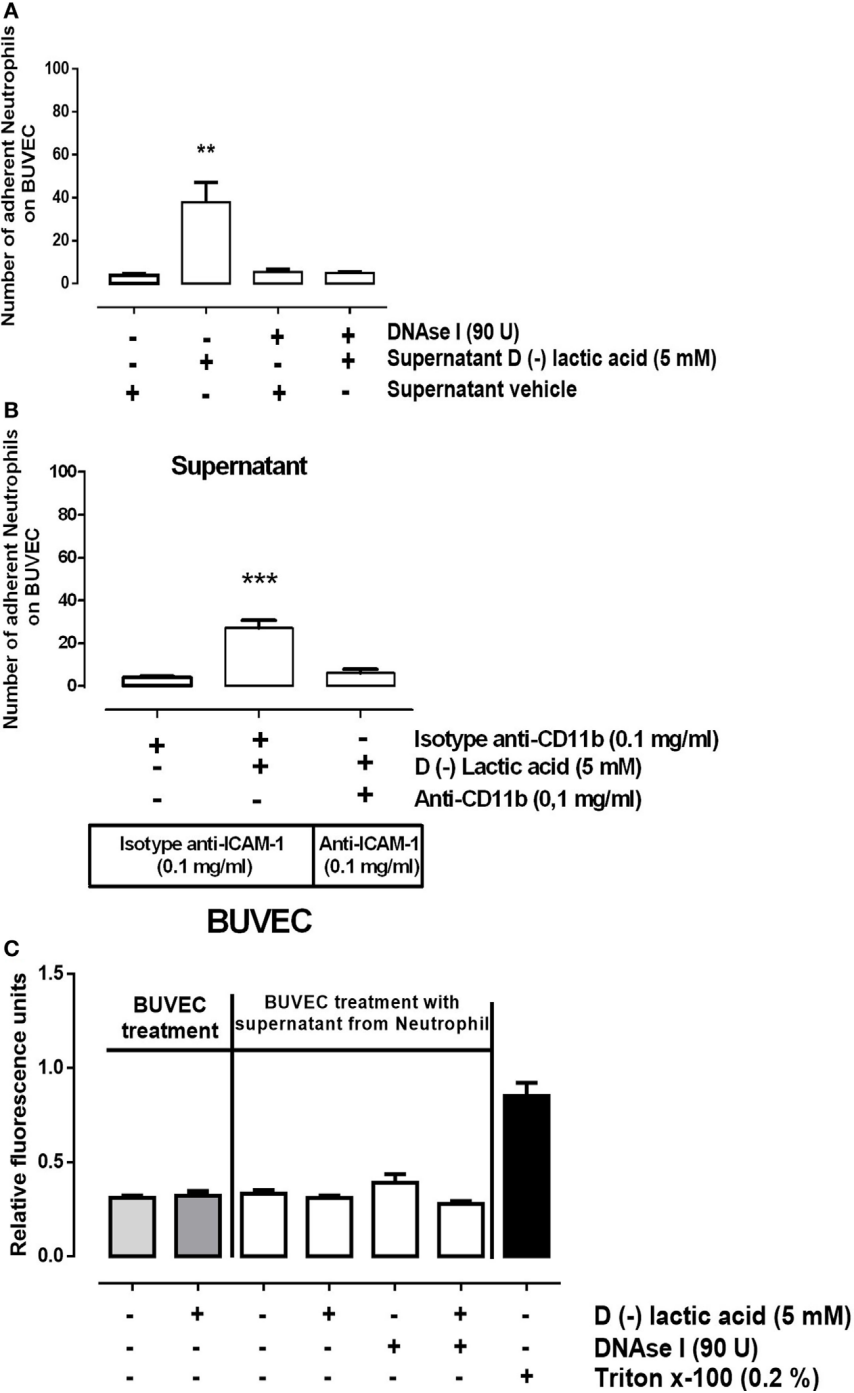
d(−) lactic acid induced the release of cell-free DNA from neutrophils that activated bovine umbilical vein endothelial cell (BUVEC) sheets by ICAM display. **(A)** Supernatants collected from neutrophils treated with 5 mM d(−) lactic acid plus or minus DNase I were perfused onto BUVEC for 10 min. Next, fresh, untreated neutrophils were perfused on endothelial monolayers, *n* = 5, **P < 0.01 compared with vehicle controls, using Dunnett’s multiple comparison test. **(B)** Supernatants collected from neutrophils treated with 5 mM d(−) lactic acid or vehicle were incubated with anti-CD11b or isotype control antibodies and then were perfused for 10 min onto BUVEC pre-incubated with anti-ICAM-1, or isotype control antibodies. Fresh neutrophils were then perfused onto the prepared BUVEC sheets, *n* = 5, ***P < 0.001 compared with vehicle controls, using Dunnett’s multiple comparison test. **(C)** Viability was measured of BUVEC treated with 5 mM of d(−) lactic acid, supernatant from neutrophils treated with 5 mM d(−) lactic acid plus or minus DNase I, or vehicle (0.01% ethanol) for 30 min at 37°C. Positive controls for cell death were obtained using 0.2% of Triton X-100, *n* = 5.

Previously, d(−) lactic acid stimulation of bovine neutrophils resulted in the release of NET, suggesting that NET may participate in neutrophil adhesions onto activated endothelial cell sheets. cf-DNA may function to activate endothelial cells, which could promote firm neutrophil adhesions. To test this, supernatants from neutrophils previously stimulated with 5 mM d(−) lactic acid or vehicle were collected in the presence or absence of DNase I. Supernatants were perfused on bovine endothelial monolayers in parallel plate flow chambers for 10 min. Next, untreated neutrophils were perfused into pre-treated chambers and adhesion assays were performed. Adhesions of freshly infused, unstimulated neutrophils were significantly increased when endothelial cell sheets were exposed to supernatants collected from 5 mM d(−) lactic-stimulated neutrophils (Figure 5A). Conversely, decreased neutrophil–endothelium adhesions were stimulated by supernatant collected from neutrophils treated with both 5 mM d(−) lactic acid and anti-CD11b antibodies. Endothelial cell sheets were previously blocked with anti-ICAM-1 antibodies or control antibody isotypes (Figure 5B). Together, these data suggested that d(−) lactic acid neutrophil stimulation mediated release of soluble NET structures. These soluble components enabled neutrophil adhesions onto endothelia through a CD11b/ICAM-1-dependent mechanism. Neither supernatant affected endothelial cell viabilities (Figure 5C).

## Discussion

The molecular mechanism underpinning neutrophil adhesion is a complex multistep process, which involves selectin and integrin proteins (63). Here, we tested a potential role of d(−) lactic acid, a metabolite associated with aseptic inflammatory process, in mediating neutrophil to endothelial sheet adhesion. We observed that d(−) lactic acid induced significant neutrophil adhesion to endothelial cells under physiological flow conditions *in vitro*. Interestingly, adhesion was exclusively observed when neutrophils were induced by d(−) lactic acid, and not when BUVEC monolayers were stimulated alone. By contrast, TNFα induced significant neutrophil adhesion regardless of whether neutrophils or BUVEC were stimulated. Previous evidence has shown that TNFα increased CD11b [CR3 or MAC-1 (64)] and ICAM-1-expressions [CD54 (65)] in neutrophils and endothelia, respectively. TNFα neutrophil-induced adhesion on untreated endothelia was described as a transient effect under flow conditions (66).

We demonstrated through use of anti-ICAM-1 antibodies either alone, or concomitant with anti-CD11b antibodies, that d(−) lactic-stimulated neutrophil adhesion onto bovine endothelial sheets was significantly reduced under physiological flow conditions, suggesting that adhesion was a CD11b/ICAM-1-dependent interaction. CD11b mediated attachment of neutrophils onto endothelial cell sheets after stimulation with Platelet activating Factor under flow conditions (67). Furthermore, the use of antibodies against the CD11b/CD18 complex reduced neutrophil adhesion to endothelial sheets (66, 68, 69). During lactic acidosis, an upregulation of CD11b/CD18 expression in human neutrophils has been observed (40). Consistent with these findings in humans, in bovine isolates 10 mM of d(−) lactic neutrophil stimulation increased CD11b cell surface expression (42). Because, cell surface CD11b on neutrophils bound to endothelial-expressed ICAM-1 (70), it has been suggested that CD11b/ICAM-1 binding is critical for pro-inflammatory agents-mediated neutrophil adhesions. Interestingly, neutrophil recruitments into sinusoidal capillaries of the liver required only CD11b–ICAM-1 interactions for firm adhesion; neutrophil selectin-mediated rolling was unnecessary (71). This process could mediate aseptic neutrophil recruitment observed during d(−) lactic acidosis in cattle (38).

d(−) lactic acid-induced NET was also bound to CD11b and citrullinated H_4_. NETosis has been involved in pathogenesis of aseptic inflammatory processes (19, 22). So far, a myriad of stimuli have been described to induce NETosis (72). NET structures induced by these agents are composed of chromatin backbones (DNA and histones) covered in proteins with proteolytic and antibacterial functions, such as myeloperoxidase [MPO (73)], neutrophil elastase (5), cathelicidins such as LL-37, proteinase 3, cathepsin, lactoferrin, and gelatinase (72, 74). To the best of our knowledge, the present report is the first demonstrating the presence of CD11b on extracellular NET structures induced by d(−) lactic acid. The precise role of CD11b on NET requires further investigation, but it might involve adhesion of NET structures onto endothelial walls of blood vessels *in vivo*.

It was previously described that hyper-citrullination of histones (citrullinated H_4_) was required for nuclear chromatin decondensation and NET release *via* PAD4 (15, 16). Using Cl-amidine, a PAD4 inhibitor, d(−) lactic acid-induced NETosis was significantly inhibited in our models, thus indicating it to be a PAD4-dependent mechanism. In a similar way, Cl-amidine reduced histone H_4_ citrullination 3 and NETosis in neutrophils from a sepsis murine model (75). Moreover, superoxide and hydrogen peroxide induced NET formation (16, 76). Thus, in neutrophils derived from PAD4 knockout murine models, hydrogen peroxide was unable to induce NET release and proper histone citrullination (16). This suggested a key role of NAPDH oxidase in this effector mechanism. Supporting this, diphenyleneiodonium (DPI), a potent NAPDH oxidase inhibitor, significantly decreased NET release and citrullination of H_3_ histone (76). We previously reported that d(−) lactic acid exposure of bovine neutrophils failed to induce ROS production (42), suggesting that citrullination of H_4_ and d(−) lactic acid-induced NET release may be NADPH oxidase independent. Interestingly, neutrophils treated with nicotine (77) or Ca^2+^ ionophore (78) produced extensive histone citrullination and NET formation, in an NADPH oxidase-independent manner. PAD4 has been widely implicated in DNA decondensation and NET release in neutrophils (15). However, other mechanisms could contribute to this phenomenon. For example, d(−) lactic acid in cervical human cell lines inhibited histone deacetylase, favoring the decondensation of chromatin through a PAD4-independent mechanism (79).

We identified MCT1 and MCT4 on bovine neutrophil membranes. MCT1 may be responsible for d(−) lactic acid uptake, given that MCT4 has mainly been implicated in monocarboxylate efflux (80). In support of this hypothesis, Ar-c155858, a specific MCT1 inhibitor, significantly reduced intracellular levels of d(−) lactic acid. MCT1 has previously been associated with uptake of this class of organic acid, rather than its extrusion, which is dependent on metabolism, cell type, and substrate concentrations between the extracellular and intracellular spaces (81, 82). In monocytes, lactic acid uptake is dependent on MCT1, activating intracellular signaling involved in the expression of COX-2 (83). Our experiments suggested that inhibition of d(−) lactic acid uptake *via* MCT1 reduced neutrophil adhesion onto endothelial cell sheets. We detected CD147 in bovine neutrophils, this molecule has been closely tied to membrane expression of MCT1 and MCT4 (59) and could participate in chemotaxis and adhesion processes in human neutrophils (84, 85). Evidence was also generated using pharmacological inhibitors of MCT1 and by silencing MCT1 in HeLa cells, which decreased migration in glucose-starving conditions can occur (86). The current study provides the first evidence suggesting a role for MCT1 in leukocyte cell adhesion. In addition, Ar-c155858 also impaired d(−) lactic-triggered NET formation in bovine neutrophil models, suggesting that adhesion of the cell types was d(−) lactic acid dependent and occurred after uptake of this organic acid and release of NET. Indeed, addition of Cl-amidine and DNase I each significantly reduced bovine neutrophil adhesion onto vascular endothelial sheets under flow conditions. Neither Ar-c155858 nor DNase I affected neutrophil adhesion after TNFα stimulation, demonstrating specificity for the d(−) lactic acid-induced pathway. Recent evidence proposes that cf-DNA levels elevated in the blood stream during sepsis are surrogate markers of circulating NET, which correlated with severity of sepsis and organ failure in humans (87). We observed that supernatants of neutrophils previously treated with d(−) lactic acid, enhanced adhesion of untreated neutrophils, which was not observed when treated with d(−) lactic acid alone. Moreover, DNase I treatment reduced the adhesion induced by supernatants, supporting a role for cf-DNA in downstream adhesion of neutrophils to endothelia after d(−) lactic acid exposure.

The presence of histone-bound DNA extracellular traps after d(−) lactic acid exposure to neutrophils may also affect endothelial cell viability. Exposure to neither d(−) lactic acid nor stimulated neutrophil supernatants influenced endothelial cell viability in our models. The role of NETosis on endothelial cells remains unclear. However, NET have mediated endothelial cell damage and dysfunction in inflammatory processes (88, 89). The presence of extracellular histones has induced endothelial cell damage (90, 91). Circulating soluble thrombomodulin, a marker of endothelial cell damage, was significantly increased in mice shortly after the injection of histones (75 mg/kg), suggesting a rapid endothelial cell damage mechanism (90, 91). Discrepancies between these data and our findings could be attributed to the concentration of histone in the NET released by d(−) lactic acid stimulation, or the responses could differ in two different model systems. It remains a possibility that we observed direct vascular damage induced by neutrophils (92), which is a pathological finding in ruminal acidosis (93). Here, we demonstrated that d(−) lactic acid-treated neutrophil supernatant-induced neutrophil adhesions were significantly attenuated by incubation of neutrophils with anti-CD11b and endothelial cell sheets with blocking anti-ICAM-1 antibodies. These data suggested that d(−) lactic acid induced cf-DNA release from neutrophils and was sufficient to stimulate neutrophil adhesions onto vascular endothelia *via* CD11b/ICAM-1.

Neutrophils are known to mediate the damage of several tissues in animals suffering acute ruminal acidosis (93) supporting the regulatory capabilities of these pro-inflammatory reactions. During ruminal acidosis, both septic (33, 94, 95) or aseptic inflammatory processes (37, 38) have been observed, leading to laminitis or polysynovitis. In conclusion, the results presented here suggest a contribution of d(−) lactic acid-triggered NETosis to a myriad of pathological entities associated with ruminal acidosis in cattle.

## Ethics Statement

This study was carried out in accordance with the recommendations of “Comisión Nacional de Investigación Científica y Tecnológica,” and according to the current Chilean Animal Protect Laws, by the ethical committee of the Universidad Austral de Chile. The protocol was approved by the ethical committee of the Universidad Austral de Chile (permit number: 217/2015).

## Author Contributions

RB, CH, MH, and AT designed the project and experiments. PA, CM, IC, MC, TM-C, and LS carried out most of the experiments. RB, CH, and PA wrote the manuscript. RB and PA carried out statistical analysis and prepared figures. All authors reviewed the manuscript.

## Conflict of Interest Statement

The authors declare that the research was conducted in the absence of any commercial or financial relationships that could be construed as potential conflicts of interest.
