# Supplementary material for: d(−) Lactic Acid-Induced Adhesion of Bovine Neutrophils onto Endothelial Cells Is Dependent on Neutrophils Extracellular Traps Formation and CD11b Expression
Source: Front Immunol. 2017 Aug 15;8:975. doi: 10.3389/fimmu.2017.00975 (PMC5559443; doi:10.3389/fimmu.2017.00975)
Supplement: Supplementary file 1 [file Data_Sheet_1.DOCX]

Supplementary Material

**D (-) lactic acid-induced adhesion of bovine neutrophils onto endothelial cells is dependent on Neutrophils extracellular traps formation and CD11b expression**

Pablo Alarcón^1^, Carolina Manosalva^1, 2^, Ivan Conejeros^1^, María D. Carretta^1^, Tamara Muñoz-Caro^3^, Liliana M. Silva^3^, Anja Taubert^3^, Carlos Hermosilla^3^, María A. Hidalgo^1^, Rafael A. Burgos^1*^

^1^Institute of Pharmacology and Morphophysiology, Faculty of Veterinary Sciences, Universidad Austral de Chile, Chile,

^2^Institute of Pharmacy, Faculty of Sciences, Universidad Austral de Chile, Chile,

^3^Institute of Parasitology, Faculty of Veterinary Medicine, Justus Liebig University Giessen, Germany

*** Correspondence:** Corresponding Author: [rburgos1@uach.cl](mailto:rburgos1@uach.cl)


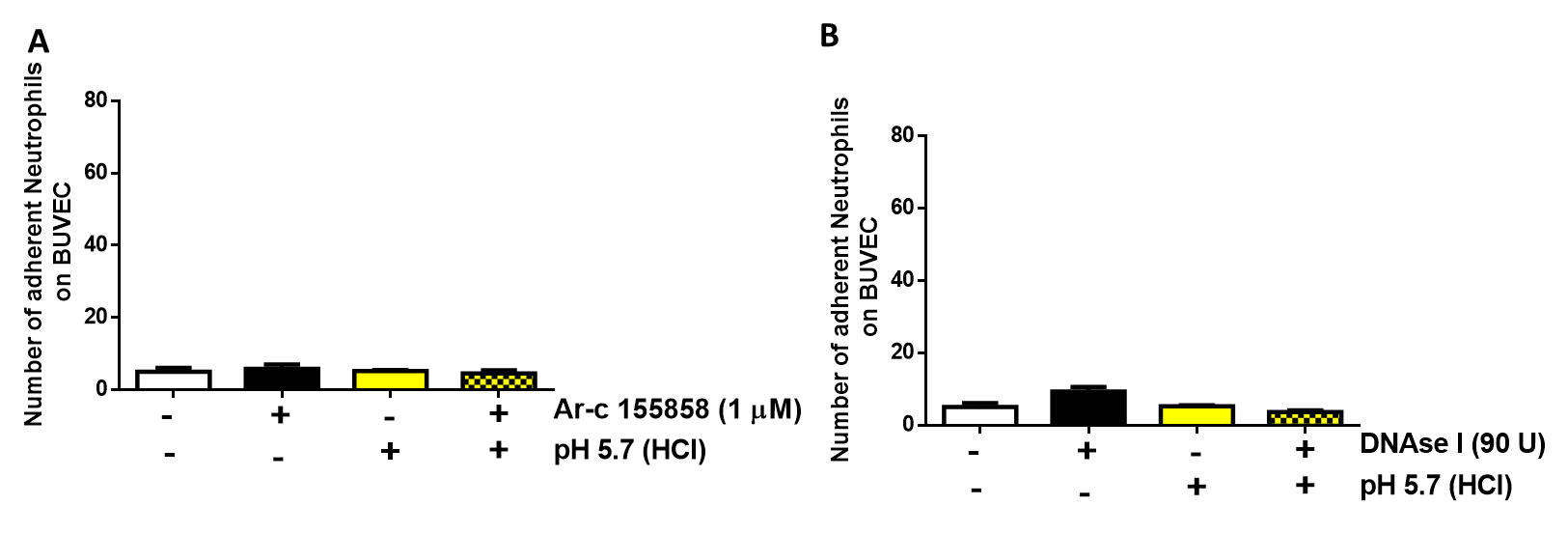


**Supplementary Figure 1.** pH acid no increase the adhesion of neutrophil onto endothelium. Mean ± S.E.M. of adherent neutrophils on BUVEC, treated with 1 µM Ar-c 155858 for 1 h (A) and then stimulated with HCl (final pH 5.7) in concomitant or not with 90 U DNAse I (B)

**
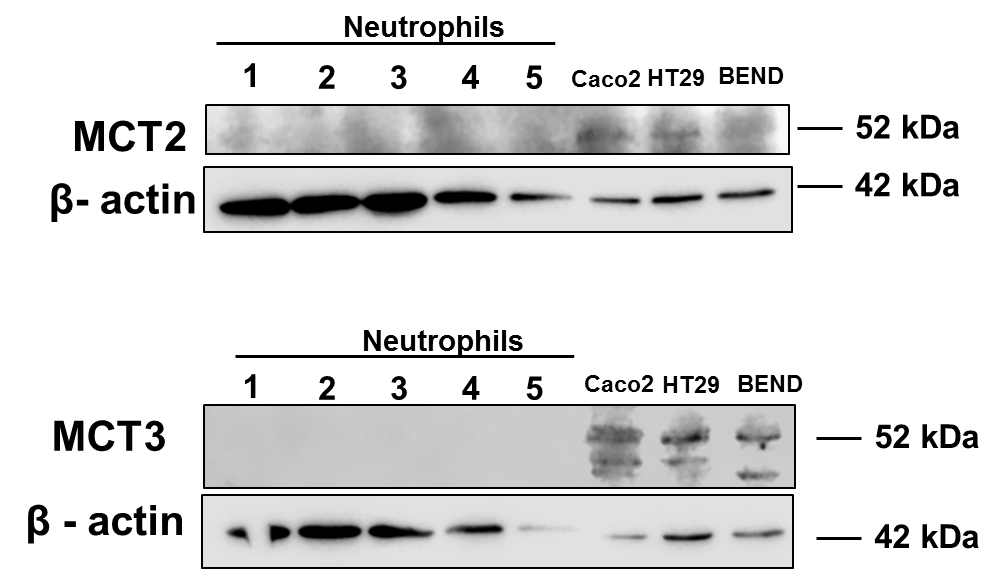
**

**Supplementary Figure 2.** MCT2 and MCT3 are not presence on neutrophils bovine. Western blot of MCT2 and MCT3 of lysed from 5 different neutrophils isolates, using Caco2, HT29 and BEND cells as a positives controls and normalized with beta-actin protein
